# Comparison of long-term outcome between hemicolectomy and partial colectomy in the elderly: a large population-based study

**DOI:** 10.18632/oncotarget.16993

**Published:** 2017-04-10

**Authors:** Xu Guan, Hanqing Hu, Wei Chen, Zheng Jiang, Zheng Liu, Zhixun Zhao, Yinggang Chen, Guiyu Wang, Xishan Wang

**Affiliations:** ^1^ Department of Colorectal Surgery, The Second Affiliated Hospital of Harbin Medical University, Harbin, China; ^2^ Department of Colorectal Surgery, Cancer Institute & Hospital, Chinese Academy of Medical Sciences, Peking Union Medical College, Beijing, China; ^3^ Follow Up Center, The Second Affiliated Hospital of Harbin Medical University, Harbin, China

**Keywords:** colon cancer, surgery, colectomy, elderly, survival

## Abstract

Due to large progress has been achieved in surgical techniques, anesthesia and perioperative care, it is accepted that the very elderly colon cancer (CC) patient is not contraindication for surgery. However, it is a controversy that an extended or a less aggressive surgical approach should be performed for this population. Here, we identified 28110 CC patients aged ≥80 from Surveillance, Epidemiology, and End-Results (SEER) database. The surgical approaches included extended hemicolectomy (HC) and partial colectomy (PC). 5-year cancer specific survival (CSS) was obtained. Kaplan–Meier methods and Cox regression models were used to assess the correlations between prognostic factors and long-term survival. The 5-year CSS for patients treated with HC were 45.6%, which were similar to patients who received PC (44.8%), the survival difference has no statistical significance (P=0.087). The result following propensity score matching further confirmed long-term survival were equal between HC and PC. However, patients in AJCC T3/T4 stage and with tumor size ≥5cm could obtain survival benefit from the extended surgery. In conclusion, most of elderly CC patients could not obtain survival benefit from extended resection. Partial colectomy should also be considered as an alternative approach for this group of patients.

## INTRODUCTION

With aging, the incidence and prevalence of cancer increases [1]. Colorectal cancer (CRC) is by far one of the most commonly diagnosed cancers in the elderly and the incidences expected to annually increase because of the higher life expectancy [2]. The evidence has indicated that about 60% of CRC patients are over 70 years of age at the time of diagnosis, and 43% are over 75, and the highest risk of being diagnosed with CRC is between the age of 80 and 89 years [2]. Therefore, the selection of optimal treatment is very necessary and urgent for this population, especially for patients aged ≥80.

Surgical resection is still play the leading role in the curative treatment of colon cancer (CC) patients [3, 4]. The surgical approach and the extent of lymphadenectomy for CC depend on tumor location, extent of lymphatic spread and oncologic outcome. Due to improvements of surgical equipment, anesthesia and perioperative care, the surgical approaches have been made feasible and effective for the vast majority of CC patients [5]. However, because of the fragile body condition and various types of coexisting medical or psychosocial problems, the elderly CC patients are still presenting with a large challenge for most of surgeons [6].

Currently, although substantial improvements of long-term survival have been achieved in younger CC patients owing to the extended surgical approach [7], the potential influence of a more aggressive surgical procedure on survival in the very elderly CC patients has not been well defined [8]. More aggressive surgical approach could lead to larger strike for elderly CC patients compared with the younger patients who received the same surgical treatment. Therefore, reasonable surgical approach should be carefully weighed for this group of patients. The aims of this study were to establish for the first time to compare the long-term survival benefit between partial colectomy (PC) and extended hemicolectomy (HC) for CC patients aged ≥80 based on a large-scale national cohort study. In this work, we firstly compared the proportion of different surgical approaches in different age groups. Secondly, lymph nodes evaluations including the median number of lymph node and the rate of node positivity were compared between PC and HC. Thirdly, the long-term survival was compared between PC and HC for patients aged ≥80. Fourthly, we divided patients into 18 subgroups based on different demographic and clinicopathological characteristics to further determine the prognostic consistency between these two surgical approaches.

## RESULTS

### Patient characteristics

A total of 28110 eligible CC patients age ≥80 were collected during the 10-year study period, including 9352 patients underwent PC and 18758 patients underwent HC. Of the cohort, the proportion of male patients was 63.0%, white patients was 86.9%, accounting for the majority of patients collected. For patients in PC group, 25.0% of patients were in stage I, 43.0% in stage II and 32.0% in stage III; the proportions were similar to patients in HC group. 56.9% of patients were left sided CC, this proportion markedly decreased to 11.4% in HC group. Tumor in grade I/II accounted for 80.3% in PC group, which was obviously higher than HC group (72.4%). In both groups, patients in T3/T4 stage and N0 stage accounted for larger proportions; the proportions of patient with adenocarcinoma were 89.7% and 85.3% separately. The detailed information was listed in Table 1.

**Table 1:** Characteristics among patients aged ≥80

Characteristics	Partial colectomy (N=9352)		Hemicolectomy (N=18758)		P
**Gender**					<0.001
**Male**	3663	39.2%	6749	36.0%	
**Female**	5689	60.8%	12009	64.0%	
**Race**					<0.001
**Black**	544	5.8%	1229	6.6%	
**White**	7971	85.3%	16458	87.7%	
**Others**	837	8.9%	1071	5.7%	
**AJCC Stage**					0.002
**Stage I**	2338	25.0%	4327	23.1%	
**Stage II**	4021	43.0%	8275	44.1%	
**Stage III**	2993	32.0%	6156	32.8%	
**AJCC T stage**					<0.001
**T1/T2**	2641	28.2%	4905	26.1%	
**T3/T4**	6711	71.8%	13853	73.9%	
**AJCC N stage**					<0.001
**N0**	6359	68.0%	12602	67.2%	
**N1**	2042	21.8%	3902	20.8%	
**N2**	951	10.2%	2254	12.0%	
**Grade**					<0.001
**Grade I/II**	7514	80.3%	13595	72.4%	
**Grade III/IV**	1623	17.4%	4739	25.3%	
**Unknown**	215	2.3%	424	2.3%	
**Tumor location**					<0.001
**Right sided colon cancer**	4035	43.1%	16619	88.6%	
**Left sided colon cancer**	5317	56.9%	2139	11.4%	
**Histology**					<0.001
**Adenocarcinoma**	8386	89.7%	15995	85.3%	
**Mucous Tumor**	900	9.6%	2571	13.7%	
**Others**	66	0.7%	192	1.0%	
**Tumor size (cm)**					<0.001
**0-5**	5511	58.9%	9850	52.5%	
**≥5**	3841	41.1%	8908	47.5%	

**Figure 1:**
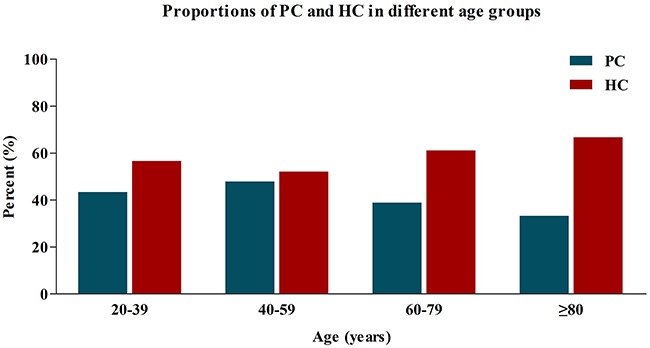
Proportions of PC and HC in different age groups PC, partial colectomy; HC, hemicolectomy.

In addition, with the aim of observing whether the selection of surgical procedure was varied in different age groups, we compared the proportions of surgical treatment strategies in different age groups of CC patients, including 2519 patients aged 20-39, 29265 patients aged 40-59, 60809 patients aged 60-79, these patients were also collected from Surveillance, Epidemiology, and End-Results (SEER) database according to the same exclusion criteria as patients aged ≥80. All these patients were then divided into HC group and PC group separately. In Figure 1, we observed an increasing proportion of patients underwent HC as age increasing. The proportion of HC was up to 66.7% for patients aged ≥80, which was obviously higher than other younger patient groups. This result also indicated that the very elderly CC patients were more likely to undergo the extended surgical procedure compared with younger patients.

### Lymph node comparisons between HC and PC

**Figure 2:**
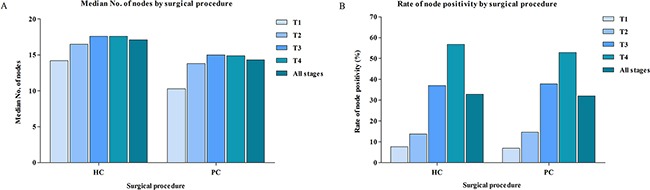
**(A)** Comparisons of median No. of lymph node between PC and HC. **(B)** Comparisons of rate of node positivity between PC and HC. PC, partial colectomy; HC, hemicolectomy.

Patients who underwent HC had a median number of 17.1 lymph nodes, and patients who underwent PC had 14.3 lymph node. In addition, we further evaluated the median of lymph nodes examined in different T stage separately (Figure 2A). With the T stage increased, the median of lymph nodes examined were increased from 14.2 to 17.6 in patients treated with HC, from 10.3 to 15.0 in patients treated with PC. Although the increased number of lymph nodes examined by HC, the rate of node positivity for patients treated with HC was 32.8%, which was similar to patient treated with PC (32.0%) (Figure 2B). The association between rate of node positivity and T stage was also presented with positive correlation.

### Survival comparison between patients in HC group and PC group

**Figure 3:**
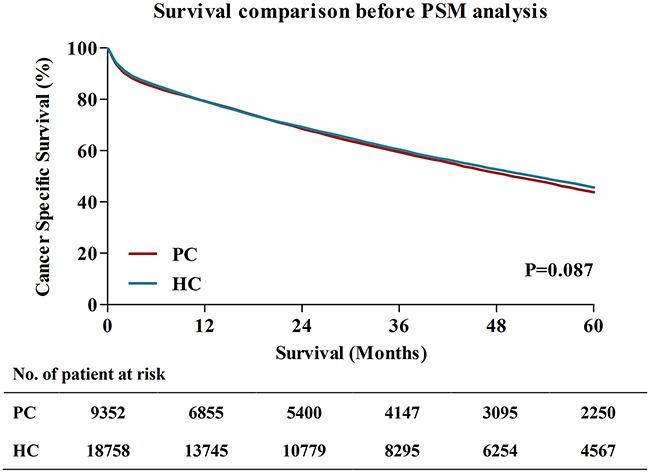
Survival comparisons between PC and HC before PSM analysis PC, partial colectomy; HC, hemicolectomy; PSM, Propensity score matching.

With the aim of estimating whether CC patients aged ≥80 could obtain survival benefit from the extended surgical procedure, we compared 5-year cancer specific survival (CSS) between patients underwent HC and patients underwent PC (Figure 3). The results showed that the 5-year CSS for patients who underwent HC were 45.6%, which were similar to patients who received PC (44.8%), the survival difference has no statistical significance (P=0.087).

**Figure 4:**
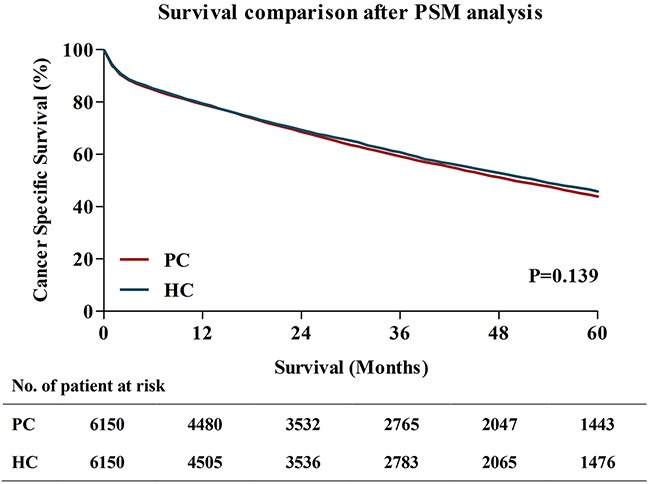
Survival comparisons between PC and HC after PSM analysis PC, partial colectomy; HC, hemicolectomy; PSM, Propensity score matching.

To reduce possible bias between two surgical groups to a minimum, the propensity score matching (PSM) analysis was performed. After PSM, there were totally 12300 patients left, with 1:1 ratio in HC group and PC group. The characteristics between two groups were well balance in the aspect of gender, AJCC stage, AJCC T stage, AJCC N stage, grade, tumor location, histology and tumor size, with P>0.05. Table 2 showed the changes of all characteristics after PSM. Then, the 5-year CSS was compared between patients in HC group and patients in PC group. The results were similar to the primary survival comparisons before PSM. The 5-year CSS for patients treated with HC were 45.8%, which was only a litter higher than those received PC (44.8%) (P=0.139) (Figure 4).

**Table 2:** Characteristics among patients aged ≥80 after propensity score matching

Characteristics	Partial colectomy (N=9352)		Hemicolectomy (N=18758)		P
**Gender**					0.055
**Male**	2251	36.6%	2354	38.3%	
**Female**	3899	63.4%	3796	61.7%	
**Race**					<0.001
**Black**	354	5.8%	457	7.4%	
**White**	5292	86.0%	5298	86.2%	
**Others**	504	8.2%	395	6.4%	
**AJCC Stage**					0.914
**Stage I**	1471	23.9%	1451	23.6%	
**Stage II**	2741	44.6%	2753	44.8%	
**Stage III**	1938	31.5%	1946	31.6%	
**AJCC T stage**					0.164
**T1/T2**	1652	26.9%	1584	25.8%	
**T3/T4**	4498	73.1%	4566	74.2%	
**AJCC N stage**					
**N0**	4212	68.5%	4204	68.4%	
**N1**	1311	21.3%	1379	22.4%	
**N2**	627	10.2%	567	9.2%	
**Grade**					0.648
**Grade I/II**	4757	77.3%	4716	76.7%	
**Grade III/IV**	1262	20.6%	1304	21.2%	
**Unknown**	131	2.1%	130	2.1%	
**Tumor location**					1
**Right sided colon cancer**	4030	65.5%	4030	65.5%	
**Left sided colon cancer**	2120	34.5%	2120	34.5%	
**Histology**					0.217
**Adenocarcinoma**	5370	87.4%	5433	88.4%	
**Mucous Tumor**	734	11.9%	673	10.9%	
**Others**	46	0.7%	44	0.7%	
**Tumor size (cm)**					0.084
**0-5**	3462	56.3%	3557	57.8%	
**≥5**	2688	43.7%	2593	42.2%	

### 18 subgroup analyses

**Figure 5:**
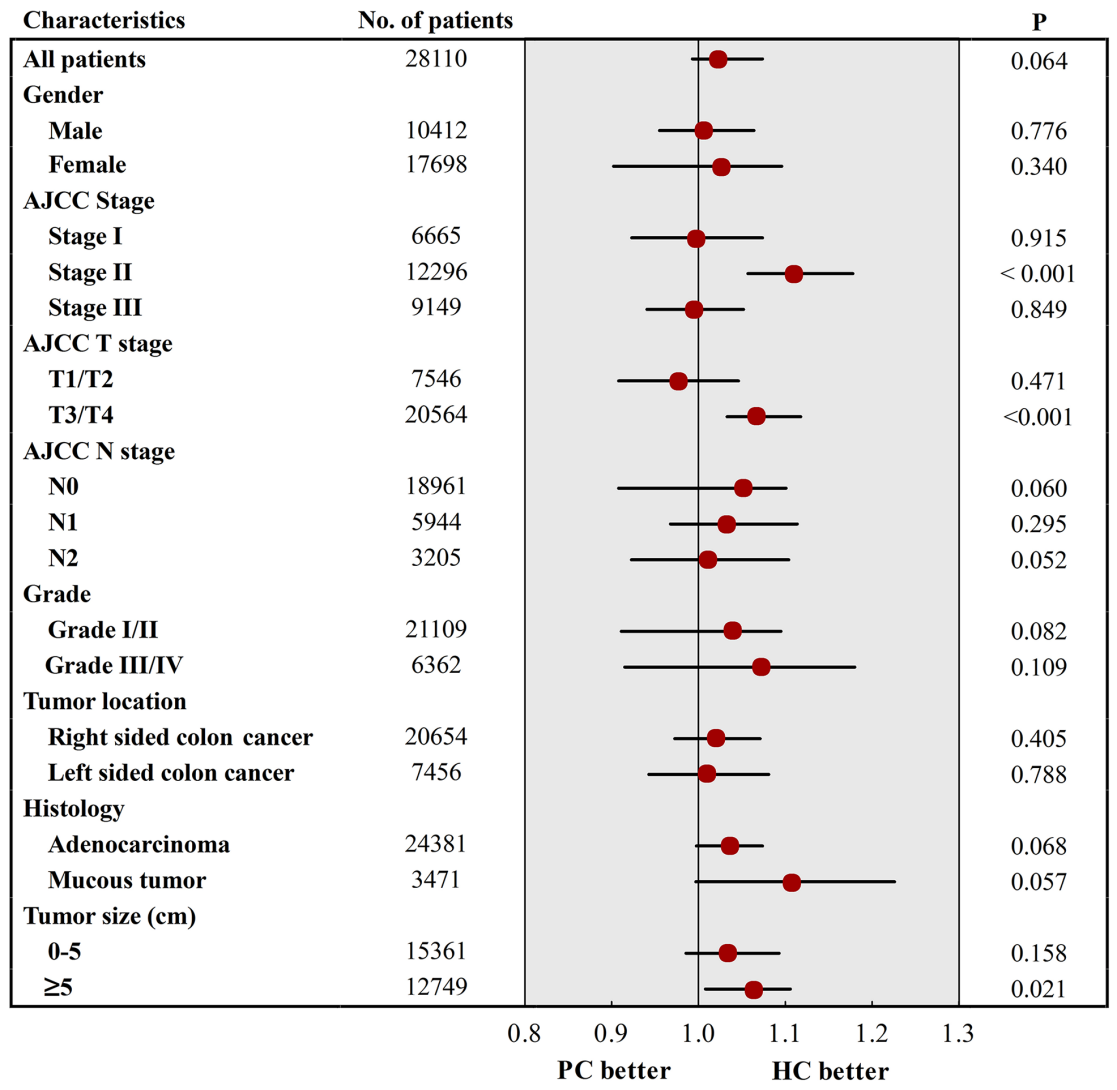
Survival comparisons between PC and HC in 18 subgroup analysis PC, partial colectomy; HC, hemicolectomy.

To further compare the prognostic consistency between patients in HC group and patients in PC group, we divided patients into 18 subgroups based on each of different demographic and clinicopathological characteristics, and Cox's regression model was separately used to estimate hazard rate (HR) and 95% confidence intervals (CIs) in each subgroup (Figure 5). The results indicated that patients who underwent HC could not obtain much more survival benefits than patients treated with PC. The influence of treatment strategy with respect to CSS was homogeneous in 15 subgroups with P>0.05. However, for patients in AJCC T3/T4 stage and with tumor size ≥5cm, they could obtain survival benefit from the extended surgical procedure. Therefore, this finding sufficiently established that the very elderly patients treated with HC not showed better long-term survival as compared with patients underwent PC, except for patient in T3/T4 and large tumor size.

### Identifying adverse prognosis factors for CC patients aged ≥80

To further explore the factors that influenced long-term survival of patients aged ≥80, univariate and multivariate Cox regression analyses were performed to determine prognostic factors (Table 3). The results suggested that patients who underwent PC were not considered as independent adverse prognostic factor for CSS in patients aged ≥80. However, other characteristics including male, black, stage II/III, stage T3/T4, stage N1/N2, grade III/IV and tumor size ≥5cm were all identified as independent poor prognostic factors.

**Table 3:** Univariate and multivariate analyses for patients aged ≥80

Characteristic		Univariate analysis		Multivariate analysis	
		HR [95% CI]	P	HR [95% CI]	P
**Gender**	Female	1	<0.001	1	<0.001
	Male	1.169 [1.132-1.208]		1.184 [1.145-1.224]	
**Race**	White	1	<0.001	1	<0.001
	Black	1.103 [1.033-1.177]		1.115 [1.045-1.191]	
	Others	0.798 [0.744-0.856]		0.755 [0.704-0.811]	
**AJCC Stage**	Stage I	1	<0.001	1	<0.001
	Stage II	1.278 [1.223-1.336]		1.396 [1.197-1.627]	
	Stage III	1.931 [1.847-2.019]		1.874 [1.785-1.972]	
**AJCC T stage**	T1/T2	1	<0.001	1	<0.001
	T3/T4	1.494 [1.438-1.553]		1.480 [1.342-1.631]	
**AJCC N stage**	N0	1	<0.001	1	<0.001
	N1	1.419 [1.365-1.476]		1.333 [1.182-1.503]	
	N2	2.166 [2.070-2.268]		1.859 [1.756-1.957]	
**Grade**	Grade I/II	1	<0.001	1	<0.001
	Grade III/IV	1.264 [1.217-1.312]		1.112 [1.069-1.157]	
**Tumor location**	Right sided colon cancer	1	0.005	1	0.296
	Left sided colon cancer	1.053 [1.016-1.092]		1.023 [0.981-1.066]	
**Histology**	Adenocarcinoma	1	<0.001	1	0.394
	Mucous Tumor	1.066 [1.017-1.118]		1.021 [0.973-1.072]	
**Tumor size (cm)**	0-5	1	<0.001	1	<0.001
	≥5	1.053 [1.016-1.092]		1.083 [1.047-1.120]	
**Surgical procedure**	Partial colectomy	1	0.087	1	0.382
	Hemicolectomy	0.964 [0.932-1.002]		0.983 [0.945-1.022]	

## DISCUSSION

In this aging population, the incidence of CRC steadily increases as age progresses, it is necessary to evaluate survival outcomes of these patients to enhance the resource allocation and future care [9]. Although almost half of the CRC were diagnosed in patients aged over 75 years, the elderly patient group is still presenting with severe disparities regarding the selection of various surgical procedures [10]. Currently, the elderly always are excluded from randomized clinical trials, which have led to a lack of evidence-based guidelines for this group of patients.

In order to face the increased comorbidities and the poor performance status for the elderly CC population, accumulating studies have attempted to explore a wide range of possible solutions to improve the long-term outcomes of this group of patient [11–15]. However, none of studies have paid attention to the influence of surgical approaches on long-term survival among the elderly patients. This is the first large population-based study that compares the long-term survival in patients aged ≥80 between PC and HC. Here, the findings showed that the very elderly CC patients were more likely to undergo HC compared with the younger patients. However, most of the elderly patients could not obtain survival benefit from extended surgical resection compared with PC.

The potential reasons for the main conclusion in this study are worthy of further discussion. Firstly, for resectable non-metastatic CC, the optimal surgical approach is colectomy with en bloc removal of the regional lymph nodes [16, 17]. The extent of colectomy and the extent of lymphadenectomy for CC depend on tumor location, extent of lymphatic spread and oncologic outcome; the main goal of surgery aiming for cure include resection of the primary tumor, the portion of the bowel and arterial arcade containing the regional lymph nodes. To evaluate oncologic safety of PC, it is essential to know whether the extended HC contain the unnecessary resection range and lymph nodes dissection that beyond the really needed resection range in elderly patients. Here, although the number of lymph nodes examined in patients treated with HC was far higher than those who underwent PC, the rate of positive lymph node was not accordingly improved, which indicated that the number of lymph nodes examined by PC was sufficient for proper staging among elderly patients. Therefore, the same rate of positive lymph node between PC and HC might partially explain why very elderly patients who underwent PC could obtain similar survival benefit from HC. Secondly, the life expectancies of patients aged ≥80 were obviously shorter than younger patients, because of the higher cardiac or pulmonary comorbidity rate, lower nutritional conditioning, and other increased frequency of comorbidity conditions. Furthermore, the metabolism of elderly patients markedly slows down, and tumor progression is not so rapid as younger patients, which probably contribute to lower occurrences of tumor recurrence and metastasis in elderly patients, especially in patients aged ≥80. Therefore, the short life expectancy and slow tumor progression could influence the prognosis in elderly patients, which may cover up the effect of surgical approach on long-term outcomes in this group of population.

Due to large progress have been achieved in surgical techniques, anesthesia and perioperative supportive care, it is currently accepted that elderly patients is not a contraindication for surgery [8]. Nevertheless, it is still hard to make a selection between a less aggressive and extended surgical approach for this elderly patient group [18, 19]. Furthermore, the selection of surgical approach, and the extent of resection needed for various tumor positions, is still unclear for the elderly patients, because these very elderly patients were not included in most of high quality studies. The general thinking has indicated that the larger extent of colon resection, the more technical difficulties associated with such surgical approaches and more chances to face the risk of postoperative complications [20]. It is well acknowledged that elderly patients have an increased risk of comorbidities that contribute to a higher rate of postoperative complications and mortality [21]. A systematic review collecting 34194 patients compared the outcomes of CC patients in different age groups [5]. The study reported that elderly patients had a higher rate of comorbidities; and they were less likely to undergo radical surgical treatment. Based on above evidence, we hence recommended PC could be selectively attempted for this group of elderly patients.

Despite of the strengths of this work including large sample size, PSM test, there still had some potential limitations in SEER database including lack of information on perioperative morbidity and comorbidities, lack of information about the quality of surgery, such as the level of vascular ligation and the degree of node dissection, lack of information regarding the adjuvant chemotherapy for CC patients and lack of detailed information associated with treatment compliance and histopathologic features including angiolymphatic invasion and margin of resection [22]. In addition, the study also has the limitation in its widespread use because of the collected patients only representing the population in the United States. Despite these limitations, SEER database remains a valuable resource to evaluate the patterns and trends in tumor features, patient characteristics, cancer therapies, and survival outcomes [23].

In conclusion, this population-based study demonstrated that patients aged ≥80 accounted for a large proportion of CC patients, they had obviously higher proportion of undergoing the extended resection compared with the younger patients. However, the elderly CC patients could not obtain survival benefit from extended resection compared to PC. Therefore, this finding might strengthen that PC should also be considered as an alternative approach for CC patient aged ≥80. Prospective randomized clinical trials specific to the elderly population are encouraged to assess the pattern of lymph node metastasis and to develop the high quality evidence-based guidelines for this group of patients.

## MATERIALS AND METHODS

### Data source

We identified the cancer cases from the SEER cancer registry [24]. The SEER is an openly accessed database, which covers approximately 28 percent of the US population. It includes the demographic, incidence and survival data from 17 population-based cancer registries. The authors could extract cancer cases and population data from SEER database. Data in the SEER database do not require informed patient consent, because they were anonymized and de-identified prior to release. We have got permission to acquire the research data file in the SEER program by National Cancer Institute, USA and the reference number was 10249-Nov2015. The study design was approved by the Ethics Committee of the Second Affiliated Hospital of Harbin Medical University.

### Study population

We obtained patients diagnosed with CC in stage I to stage III according to Site Recode classification. The collected patients were diagnosed from 2004 to 2013, because the seventh edition of AJCC stage system was available in SEER database since 2004. All patients were diagnosed at the age of more than 80 years. The surgical treatment procedures for these very elderly CC patients included two modalities. 1) HC or greater (but less than total), right or left colectomy. The HC here is the removal of total right or left colon and a portion of transverse colon; 2) PC (less than HC), such as enterocolectomy, ileocolectomy, cecectomy, partial resection of transverse colon and flexures and sigmoidectomy. Other clinical characteristics extracted from SEER database included gender, race, AJCC stage, grade, histology, tumor size and tumor location. The exclusion criteria were as follows: dead due to other causes and alive with no survival time.

### Statistical analysis

Firstly, we evaluated the differences in patient characteristics between HC group and PC group using the χ^2^ test. The CSS was defined as the time from the CC diagnosis until cancer metastasis or recurrence, cancer-associated death and the end of follow up. The CSS was estimated with Kaplan-Meier method, and log-rank tests were used to compare the differences of CSS curves. Univariate and multivariate Cox's regression model were performed to estimate HR and exact 95% CIs. Furthermore, the patients were classified into different subgroups based on different characteristics, then these subgroup analyses of CSS were separately performed using Cox regression model to further determine the prognostic consistency between HC group and PC group. All statistical tests were two sided, P<0.05 was considered to be statistical significance. All statistical analyses were estimated using the statistical software package SPSS 20.0 (IBM Corp, Armonk, NY, USA) and R version 2.12.0 (www.r-project.org).

### PSM

A propensity 1:1 matched analysis was done to reduce possible bias to a minimum in this retrospective analysis. Propensity scores were calculated using logistic regression model for each patient in both HC group and PC group. The covariates included in the regression were gender, race, AJCC stage, AJCC T stage, AJCC N stage, grade, histology, tumor size and tumor location. Patients in two groups were matched based on the propensity score. Covariates balance between two groups was examined by χ^2^ test. The survival comparisons were then performed for the propensity score-matched patients using the same methods as those in the primary analysis.
